# Supplementary material for: Gene products and processes contributing to lanthanide homeostasis and methanol metabolism in Methylorubrum extorquens AM1
Source: Sci Rep. 2020 Jul 29;10:12663. doi: 10.1038/s41598-020-69401-4 (PMC7391723; doi:10.1038/s41598-020-69401-4)
Supplement: Supplementary file 1 — Supplementary Tables [file 41598_2020_69401_MOESM1_ESM.pdf]

Supplementary information submitted to Scientific Reports

Gene products and processes contributing to lanthanide homeostasis and methanol metabolism in *Methylorubrum extorquens* AM1

Paula Roszczenko-Jasińska,[1,a, £,] Huong N. Vu,[2,a,£] Gabriel A. Subuyuj,[2,a] Ralph Valentine Crisostomo,[2,a] James Cai,[2] Nicholas F. Lien,[2] Erik J. Clippard,[2] Elena M. Ayala,[2] Richard T. Ngo,[2] Fauna Yarza,[2,a] Justin P. Wingett,[2] Charumathi Raghuraman,[2] Caitlin A. Hoeber,[2] Norma C. Martinez-Gomez,[1,*] Elizabeth Skovran[2,*]

[1]Department of Microbiology and Molecular Genetics, Michigan State University, East Lansing, USA

[2]Department of Biological Sciences, San José State University, San José, California, USA

[£] Both authors contributed equally and share first authorship. *Author order was determined both alphabetically and in order of increasing seniority.*

[*] Correspondence:
Norma Cecilia Martinez-Gomez, mart1754@msu.edu
Elizabeth Skovran, elizabeth.skovran@sjsu.edu

[a] Present address: PR-J: Institute of Microbiology, University of Warsaw, Warsaw, Poland; HNV: Department of Microbiology, University of Georgia, Athens, Georgia; USA; GAS: Department of Microbiology and Molecular Genetics, University of California at Davis, Davis, California, USA; RVC: Molecular Biology Institute, University of California at Los Angeles, Los Angeles, California, USA; FY: Department of Biochemistry and Biophysics, University of California at San Francisco, San Francisco, California, USA

Table S1. Growth parameters for strains grown in methanol medium with and without La^{3+}.

**Strain**	**Growth Rate (h^{-1})[£]**	
	**MeOH**	**MeOH La^{3+}**
**Growth of control strains**		
Wild type	0.14 ± 0.01	0.16 ± 0.01
mxaF	NG	0.16 ± 0.01
xoxF1	30 h lag, 0.09 ± 0.01	6-9 h lag, 0.07 ± 0.00
xoxF1 xoxF2	NG*	6 h lag, 0.04 ± 0.01
mxaF xoxF1 xoxF2	NG	6 h lag, 0.04 ± 0.00
**Growth of strains defective in methanol metabolism independent of La^{3+}**		
pqqBCDE	NG	NG
pqqF	NG	NG
cycK	NG	NG
ccmB	NG	NG
ccmC	NG	NG
META1_2359	NG*	NG*
META1_3908	24 h lag, 0.05 ± 0.00	15 h lag, 0.07 ± 0.00
META1p2024	9 h lag, 0.09 ± 0.01	6 h lag, 0.10 ± 0.01
META1_0863	0.13 ± 0.00	0.14 ± 0.00

[£]Data for a minimum of three biological replicates are reported.
* indicates if a suppressor mutation allowed eventual growth of the strain.
NG indicates no growth. Methanol is abbreviated as MeOH.

Table S2. Strains and plasmids used in this study.

Strain or plasmid	Description	Reference
M. extorquens		
AM1	Rif^R derivative (wild type)	[1]
CM194.1	Δ*mxaF*	[2]
ES2813	Δ*mxaF* Δ*fae*	This study
ES2432	Δ*xoxF1*	This study
ES2435	Δ*xoxF2* Δ*xoxF1*	This study
ES2439	Δ*mxaF* Δ*xoxF2* Δ*xoxF1*	This study
ES2386	*pqqBCDE::Km*	This study
ES2360	*pqqF::Km*	This study
ES3941	*cycK::Km*	This study
ES3968	*ccmB::Km*	This study
ES3965	*ccmC::Km*	This study
ES2817	Δ*xoxG*	This study
ES2821	Δ*xoxJ*	This study
ES2785	Δ*META1p0863*	This study
ES3947	*MexAM1_META1p1771::Km*	This study
ES3282	Δ*orf6*	This study
ES2781	Δ*orf7*	This study
ES3962	*MexAM1_META1p2359::Km*	This study
ES3959	*hss::Km*	This study
ES3257	*MexAM1_META1p3908::Km*	This study
ES2861	*MexAM1_META1_3909::Km*	This study
ES3950	*lutA::Km*	This study
ES4122	*lutB::Km*	This study
ES4444	*lutC::Km*	This study
ES4389	*lutD::Km*	This study
ES3953	*lutE::Km*	This study
ES4249	*lutF::Km*	This study
ES3956	*lutG::Km*	This study
ES2879	*lutH::Km*	This study
ES3983	Δ*mxaF lutA::Km*	This study
ES4124	Δ*mxaF lutB::Km*	This study
ES4447	Δ*mxaF lutC::Km*	This study
ES4392	Δ*mxaF lutD::Km*	This study
ES3986	Δ*mxaF lutE::Km*	This study

Table S2. (continued)

**Strain or plasmid**	**Description**	**Reference**
M. extorquens		
ES4246	Δ*mxaF lutF::Km*	This study
ES3989	Δ*mxaF lutG::Km*	This study
ES2962	Δ*mxaF lutH::Km*	This study
ES2231	AM1 / pCM62	This study
ES4293	*lutA::Km* / pCM62	This study
ES4295	*lutA::Km* / pES667	This study
ES5029	*lutB::Km* / pCM62	This study
ES5030	*lutB::Km* / pES810	This study
ES4301	*lutE::Km* / pCM62	This study
ES4305	*lutE::Km* / pES666	This study
ES5031	*lutF::Km* / pCM62	This study
ES5032	*lutF::Km* / pES815	This study
ES4309	*lutG::Km* / pCM62	This study
ES4315	*lutG::Km* / pES668	This study
ES1819	AM1 / pES502	[3]
ES4192	Δ*xoxF2* Δ*xoxF1* / pES502	This study
ES4194	Δ*xoxG* / pES502	This study
ES4196	Δ*xoxJ* / pES502	This study
ES3743	AM1 / pHV25	This study
E. coli		
TOP10	Cloning strain (Sm^R)	Invitrogen
S17-1	Helper strain (Tp^R, Sm^R)	[4]
**Plasmid**		
pRK2013	Conjugative helper plasmid (Km^R)	[5]
pCM184	Allelic exchange suicide vector (Km^R, Tc^R, Ap^R)	[6]
pHV2	Modified pCM184 expressing *sacB* (Km^R, Tc^R, Ap^R)	This study
pCM157	Modified pCM62 expressing *cre* (Tc^R)	[6]
pCM639	Modified pCM638 carrying IS*phoA*/hah-Tc (Tc^R)	[2]
pAP5	Modified pCM62 carrying promoter-less *venus* (Tc^R)	[5]
pCM62	Broad-host-range shuttle vector (Tc^R)	[7]
pES502	pAP5 carrying *mxa* promoter region upstream of *venus* (Tc^R)	[5]
pHV25	pAP5 carrying *lutH* promoter region upstream of *venus* (Tc^R)	This study
pES667	pCM62 carrying *lutA* downstream of P_{lac} promoter (Tc^R)	This study
pES810	pCM62 carrying *lutB* downstream of P_{lac} promoter (Tc^R)	This study
pES666	pCM62 carrying *lutE* downstream of P_{lac} promoter (Tc^R)	This study

Table S2. (continued)

Strain or plasmid	Description	Reference
**Plasmid**		
pES815	pCM62 carrying *lutF* downstream of P_{lac} promoter (Tc^R)	This study
pES668	pCM62 carrying *lutG* downstream of P_{lac} promoter (Tc^R)	This study

References

1. Nunn, D. N. & Lidstrom, M. E. Isolation and Complementation Analysis of 10 Methanol Oxidation Mutant Classes and Identification of the Methanol Dehydrogenase Structural Gene of Methylobacterium sp. Strain AM1. *J. Bacteriol.* **166**, 581–90 (1986).

2. Marx, C. J., Chistoserdova, L. & Lidstrom, M. E. Formaldehyde-detoxifying Role of the Tetrahydromethanopterin-linked Pathway in Methylobacterium extorquens AM1. *J. Bacteriol.* **185**, 7160–8 (2003).

3. Vu, H. N. *et al.* Lanthanide-dependent regulation of methanol oxidation systems in Methylobacterium extorquens AM1 and their contribution to methanol growth. *J. Bacteriol.* **198**, 1250–1259 (2016).

4. Figurski, D. H. & Helinski, D. R. Replication of an Origin-containing Derivative of Plasmid RK2 Dependent on a Plasmid Function Provided in Trans. *Proc. Natl. Acad. Sci. U. S. A.* **76**, 1648–52 (1979).

5. Skovran, E., Palmer, A. D., Rountree, A. M., Good, N. M. & Lidstrom, M. E. XoxF is Required for Expression of Methanol Dehydrogenase in Methylobacterium extorquens AM1. *J. Bacteriol.* **193**, 6032–6038 (2011).

6. Marx, C. J. & Lidstrom, M. E. Broad-host-range Cre-lox System for Antibiotic Marker Recycling in Gram-negative Bacteria. *Biotechniques* **33**, 1062–1067 (2002).

7. Marx, C. J. & Lidstrom, M. E. Development of Improved Versatile Broad-host-range Vectors for Use in Methylotrophs and Other Gram-negative Bacteria. *Microbiology* **147**, 2065–75 (2001).

Table S3. Primers used in this study.

Primer name	Sequence (5' to 3')[£]	Plasmid	Gene
**Strain construction (pCM184 backbone)**			
xoxF_1740ULEcoRI	ta**gaattc**GCCCCGATGGCAGGAATTAAAGT	pES319-320	*xoxF1*
xoxF_1740URKpnI	at**ggtacc**CTGCTTCGGCTCGTACTTCCACA		
xoxF_1740DLHpaI	ta**gttaac**GCCTGTTCTACGTCCCCACCAAC		
xoxF_1740DRSacI	ta**gagctc**CTGCACCGACGAGACCAAGAAGA		
exaF_1139ULEcoRI	gt**gaattc**CCTCCGACAGCCTACTGATGAAC	pES3-4	*exaF*
exaF_1139URKpnI	ca**ggtac**CGTCTTGGCGTCGTTGAGGATGTC		
exaF_1139DLHpaI	ca**gttaac**GCCGTTCGTGTCCAACATCAACTG		
exaF_1139DRSacI	ta**gagctc**GTTGATGCCAGCCGTCGTCTTGG		
pqqBCDE_1748ULEcoRI	ta**gaattc**TCAACATCCGCCAAGTCAGCTTC	pES369-458	*pqqBCDE*
pqqBCDE_1748URKpnI	at**ggtacc**ATATCGGGAGAGGCGTTCAGCAG		
pqqBCDE_1748DLHpaI	tata**gttaac**GACGAATGTGCAAAACCCGTTGA		
pqqBCDE_1748DRSacI	tata**gagctc**ACGTCGGTCGGATAGTCGTTGTC		
pqqF_2330ULEcoRI	ta**gaattc**ACCGTCCCGCCATCACCTATCGC	pES789-555	*pqqF*
pqqF_2330URKpnI	at**ggtacc**GTGATTCAGGCCTCGCTGCGGTC		
pqqF_2330DLHpaI	ta**gttaac**GGTGACCGGCTACCTGACCAAGG		
pqqF_2330DRSacI	ta**gagctc**GTAGCGCAGGCTGGCGATCATCT		
lysR_0863ULEcoRI	at**gaattc**CAGGCGCTGTCGAAGCATTCAAG	pES431-432	*MexAM1_META1p0863*
lysR_0863URKpnI	at**ggtacc**GGGTGTTCTCCAGCCTGAGCTTC		
lysR_0863DLHpaI	ta**gttaac**AGCGAGATGCCGACGATGGAGAG		
lysR_0863DRSacI	ta**gagctc**ATGGACGTTGGAGAGGTGGATCT		
sacB_BegNcoI	tagca**ccatgg**CGATCCTTTTTAACCCATCACATA	pHV2	
sacB_EndNcoI	tata**ccatgg**GATATCGGCATTTTCTTTTGCG		

Table S3. (continued)

Primer name	Sequence (5’ to 3’)£	Plasmid	Gene
**Strain construction (pHV2 backbone)**			
fae1_1767UBMunI	tata**caattg**CGCGGTTCATCTCGCCTATCTCA	pES498-499	*fae1*
fae1_1767UENcoI	tata**ccatgg**CCGTGCTTGTTGTTGACCAGACC		
fae1_1767DBAgeI	ctct**accggt**TTGTCACGGAGCAGCGTAAGTCG		
fae1_1767DESacI	tata**gagctc**GTGACGCTGTGGGTCGATGC		
cycK_1293UBAatII	tata**gacgtc**CCAGCCTGAAGGAACGAGAAGAAGC	pES604-605	*cycK*
cycK_1293UENcoI	tata**ccatgg**CGGGCATCACAGCTTGCACCA		
cycK_1293DBBshTI	tata**accggt**CGGTCGGCGTCAGCCAGAT		
cycK_1293DESacI	tata**gagctc**GTGACGACGATGCGCCGGAC		
ccmB_2825UBAatII	tata**gacgtc**CCATGTGGCATCACCGAATCAATGC	pES622-623	*ccmB*
ccmB_2825UENcoI	taat**ccatgg**TCAGGAAGAAGACGAGCGAGAGC		
ccmB_2825DBBshTI	tata**accggt**GCTGATGGTCCCGACTCTGATCTT		
ccmB_2825DESacI	tata**gagctc**AACCGATGTGAGGCCGATCTCC		
ccmC_2732UBAatII	tata**gacgtc**AGAGACTGAGCGGGACCAAGC	pES620-621	*ccmC*
ccmC_2732UENcoI	tata**ccatgg**CCAGTAGGTCCCCCACATCGG		
ccmC_2732DBBshTI	tata**accggt**CTCGATGCTCTACCCGCTGCTC		
ccmC_2732DESacI	atat**gagctc**GGTCGCGTGCGGGATCTG		
ABC2_2359UBAatII	atat**gacgtc**TGCGTATGCGATGGTTCCAGACAA	pES618-619	*MexAM1_META1p2359*
ABC2_2359UENcoI	tata**ccatgg**AGCGGAAACACGATGAGTGGAATCG		
ABC2_2359DBBshTI	tata**accggt**CTGGACGGCATCTCCTTCACGG		
ABC2_2359DESacI	tata**gagctc**CGAGAGCATCCCGTTGATCCGAA		

Table S3. (continued)

Primer name	Sequence (5' to 3')[£]	Plasmid	Gene
**Strain construction (pHV2 backbone)**			
25pep_3908UBBglII	ctag**agatct**GGTACACAGGTTCGCCGGTTTTC	pES467-468	*MexAM1_META1p3908*
25pep_3908UENcoI	tata**ccatgg**GCCTCGTCCTCGTCCTTCTTCTT		
25pep_3908DBAgeI	atat**accggt**CCTACAAGCCCAAGGCGATGATC		
25pep_3908DESacI	tata**gagctc**CGGTCAGGAAGACGACGAACAGG		
25mem_3909UBBglII	tata**agatct**GACCTACAAGCCCAAGGCGATGA	pES469-470	*MexAM1_META1p3909*
25mem_3909UENcoI	atat**ccatgg**CGGTCAGGAAGACGACGAACAGG		
25pep_3909DBAgeI	tata**accggt**CACGCCGATGGTGATCGCCTATG		
25pep_3909DESacI	tata**gagctc**CGCTGCCGTTCTCACTTCCCTTG		
hss_2024UBAatII	tata**gacgtc**CGTCATGGGATTAAGTGCGAAACAGG	pES616-617	*hss*
hss_2024UENcoI	atat**ccatgg**CGGCTCTTGTCGTACTCGAAATGGC		
hss_2024DBBshTI	tata**accggt**GACCTGCCACTACGCCTACCAC		
hss_2024DESacI	atat**gagctc**TGCGAAGGCCCGAACCGTAAC		
xoxG_1741UBbglII	tata**agatct**GCAACTTCATCGCCTGGGACAAC	pES459-460	*xoxG*
xoxG_1741UEKpnI	acgt**ggtacc**CGTCGTTGGTGATGTGGAAGGTC		
xoxG_1741DBAgeI	atat**accggt**GCGAGAAGGAAGACAAGCCCG		
xoxG_1741DESacI	tata**gagctc**AATCGAGCGGGTGGAATGAGAA		
xoxJ_1742UBBglII	atat**agatct**TCTTCTTGGTCTCGTCGGTGCAG	pES461-462	*xoxJ*
xoxJ_1742UEKpnI	tata**ggtacc**CGCAGCTTGACCTTCAGCTCGTC		
xoxJ_1742DBAgeI	tata**accggt**GCCACAACGAGAACGACTGGAAG		
xoxJ_1742DESacI	tata**gagctc**CTATCTCGGGCTCGGCAGCAATA		

Table S3. (continued)

Primer name	Sequence (5' to 3')£	Plasmid	Gene
**Strain construction (pHV2 backbone)**			
xoxD_1771UBAatII	tata**gacgtc**GCCATGCCGAGTTCGAGGTG	pES608-609	*MexAM1_META1p1771*
xoxD_1771UENcoI	atta**ccatgg**ACCCTTGCCCTCGGTCTTCTC		
xoxD_1771DBBshTI	atat**accggt**GTGAAGGCCAAGGCCGAGAAG		
xoxD_1771DESacI	tata**gagctc**AGTCGGATTCGCGGTTGGCTAC		
orf6_1746UBMunI	atat**caattg**GAGCAGCCGCAGATGAAGAACAC	pES493-494	*MexAM1_META1p1746*
orf6_1746UENcoI	atat**ccatgg**GTCGGTCGGATAGTCGTTGTCGT		
orf6_1746DBAgeI	atac**accggt**CCTGCCCTATGACCGCTACGTT		
orf6_1746DESacI	atat**gagctc**CCGGCATTCCAACGAGATGTAGA		
orf7_1747UBMunI	atat**caattg**TTACGGCTGGGCCTATGTGAACC	pES495-496	*MexAM1_META1p1747*
orf7_1747UENcoI	atat**ccatgg**GTGTTCTTCATCTGCGGCTGCTC		
orf7_1747DBAgeI	atat**accggt**CCAACCCGCGCTACATCAAGAAC		
orf7_1747DESacI	atat**gagctc**TGACGTTGAGGATGCCCATTACC		
lutA_1778UBAatII	tata**gacgtc**GGACGGGCTTTAACTCGGTTTCC	pES610-611	*lutA*
lutA_1778UENcoI	tata**ccatgg**GGCGAAGGTCTGCTTGGTGAAG		
lutA_1778DBBshTI	taat**accggt**GACTGGGGCGATTATGTCCCGT		
lutA_1778DESacI	tata**gagctc**CCGTCGCTCAAACAAGAAACAGATGA		
lutB_1779UBAatII	atat**gacgtc**GCAAGTTCGGCCTCAAGATCACC	pES638-639	*lutB*
lutB_1779UENcoI	atat**ccatgg**GGCCTTCTCGTTGGTGACGTAGG		
lutB_1779DBAgeI	atat**accggt**CTCTACACGACCAACGGCACCTC		
lutB_1779DESacII	atat**gagctc**CCCCCTCACTTCACCGTAATCGT		

Table S3. (continued)

Primer name	Sequence (5’ to 3’)[£]	Plasmid	Gene
**Strain construction (pHV2 backbone)**			
lutC_1780UBMunI	atat**caattg**GAGGTGATCGACAACGTGCTGGT	pES642-643	*lutC*
lutC_1780UENcoI	ata**tccatgg**GTTCTGCCGCTTCTTGGTGAGGT		
lutC_1780DBMluI	atat**acgcgt**CGGCACGATTACGGTGAAGTGAG		
lutC_1780DESacI	atat**gagctc**GCTGGCGTGGTAATGCAGGTTTT		
lutD_1781UBBglII	atat**agatct**TGTCCGAGAAGGCGATGCAAG	pES644-645	*lutD*
lutD_1781UEMunI	atat**caattg**CAAGCACCACGAGGGCAAGC		
lutD_1781DBAgeI	atat**accggt**GAGCAAGGCGAGGGCGAGTT		
lutD_1781DESacI	atgg**gagctc**ATCGAGGTGCCGAGCGTGTT		
lutE_1782UBAatII	tata**gacgtc**GTCCGAGAAGGCGATGCAAGAAC	pES612-613	*lutE*
lutE_1782UENcoI	tata**ccatgg**CGGCCCTCCTGATTGTTGTAGAGC		
lutE_1782DBBshTI	tata**accggt**CGACAAGAGAGCCGTGAAGTCCG		
lutE_1782DESacI	tata**gagctc**CAGAGCGGATATAGGGCAGAAGAGG		
lutF_1783UBAatII	tatc**gacgtc**ACCCGGCTCTACAACAATCAGGAG	pES640-641	*lutF*
lutF_1783UENcoI	atat**ccatgg**TTCTGGTGGAAGAAACGCAGCAG		
lutF_1783DBAgeI	atat**accggt**CGAATTGGTGCGTTTCGCTCTCT		
lutF_1783DESacI	atat**gagctc**TCGCAGACGTAGGAGACCGACTG		
lutG_1784UBAatII	tata**gacgtc**GCTGGTCTGGCTGTTCATCTTCG	pES614-615	*lutG*
lutG_1784UENcoI	atat**ccatgg**TAGCCCAGCGTCGTCACCTTG		
lutG_1784DBBshTI	tata**accggt**CGGACAAGGTGAGCGAGTTCAAGGA		
lutG_1784DESacI	tata**gagctc**GCAACATATTTCTGCCGTTCCGTCAA		

Table S3. (continued)

Primer name	Sequence (5' to 3')[£]	Plasmid	Gene
**Strain construction (pHV2 backbone)**			
lutH_1785UBBglII	atat**agatct**TGCCGAATGATCCCGGTAAAGTC	pES465-466	*lutH*
lutH_1785UENcoI	tata**ccatgg**CGAACGAGGTGTTGAAACGAGCA		
lutH_1785DBAgeI	atat**accggt**CCCTGCTCGCTCACCAACTTCTT		
lutH_1785DESacI	atat**gagctc**TTCGGCCTCGCTTAGATTGCGTA		
**Transcriptional reporter fusion (pAP5 backbone)**			
lutH_1785BegAclI	tata**aacgtt**GCAGCCGTCGAAGCGATTTGC	pHV25	*lutH*
lutH_1785EndEcoRI	tata**gaattc**CCGAACGAGGTGTTGAAACGAGC		
**Complementation (pCM62 backbone)**			
lutA_1778BegKpnI	atat**ggtacc**CTTATGCTTCTCTCGTTGAGGCCG	pES667	*lutA*
lutA_1778EndSacI	atcg**gagctc**ATCGCCCGGATCGCTTCC		
lutB_1779BegKpnI	tattt**ggtacc**AAGCGATCCGGGCGATGC	pES810	*lutB*
lutB_1779EndSacI	tattt**gagctc**ACCTTTCGGGACGATGCCTTAGC		
lutE_1782BegKpnI	atat**ggtacc**GAGCAAGGCGAGGGCGAGTT	pES666	*lutE*
lutE_1782EndKpnI	atat**gagctc**GGCGGGATTCGAGGGAGACC		
lutF_1783BegKpnI	tattt**ggtacc**AAGGGTCGCATCGTCGCCCGTGG	pES815	*lutF*
lutF_1783EndSacI	tattt**gagctc**ACGAGCCGTAGCCCAGCGTCG		
lutG_1784BegKpnI	atat**ggtacc**GCCGCAATCGCCTACGATCC	pES668	*lutG*
lutG_1784EndSacI	atat**gagctc**CTTCGTCGCTTCCGACCGAAAG		

[£]Restriction endonuclease sites are in bolded font. Capitalized letters represent bases that anneal to the target DNA sequence.
