# Supplementary material for: Gene products and processes contributing to lanthanide homeostasis and methanol metabolism in Methylorubrum extorquens AM1
Source: Sci Rep. 2020 Jul 29;10:12663. doi: 10.1038/s41598-020-69401-4 (PMC7391723; doi:10.1038/s41598-020-69401-4)
Supplement: Supplementary file 2 — Supplementary Figures [file 41598_2020_69401_MOESM2_ESM.pdf]

Supplementary information submitted to Scientific Reports

Gene products and processes contributing to lanthanide homeostasis and methanol metabolism in *Methylorubrum extorquens* AM1

Paula Roszczenko-Jasińska,[1,a, £,] Huong N. Vu,[2,a,£] Gabriel A. Subuyuj,[2,a] Ralph Valentine Crisostomo,[2,a] James Cai,[2] Nicholas F. Lien,[2] Erik J. Clippard,[2] Elena M. Ayala,[2] Richard T. Ngo,[2] Fauna Yarza,[2,a] Justin P. Wingett,[2] Charumathi Raghuraman,[2] Caitlin A. Hoeber,[2] Norma C. Martinez-Gomez,[1,*] Elizabeth Skovran[2,*]

[1]Department of Microbiology and Molecular Genetics, Michigan State University, East Lansing, USA

[2]Department of Biological Sciences, San José State University, San José, California, USA

[£] Both authors contributed equally and share first authorship. *Author order was determined both alphabetically and in order of increasing seniority.*

[*] Correspondence:
Norma Cecilia Martinez-Gomez, mart1754@msu.edu
Elizabeth Skovran, elizabeth.skovran@sjsu.edu

[a] Present address: PR-J: Institute of Microbiology, University of Warsaw, Warsaw, Poland; HNV: Department of Microbiology, University of Georgia, Athens, Georgia; USA; GAS: Department of Microbiology and Molecular Genetics, University of California at Davis, Davis, California, USA; RVC: Molecular Biology Institute, University of California at Los Angeles, Los Angeles, California, USA; FY: Department of Biochemistry and Biophysics, University of California at San Francisco, San Francisco, California, USA

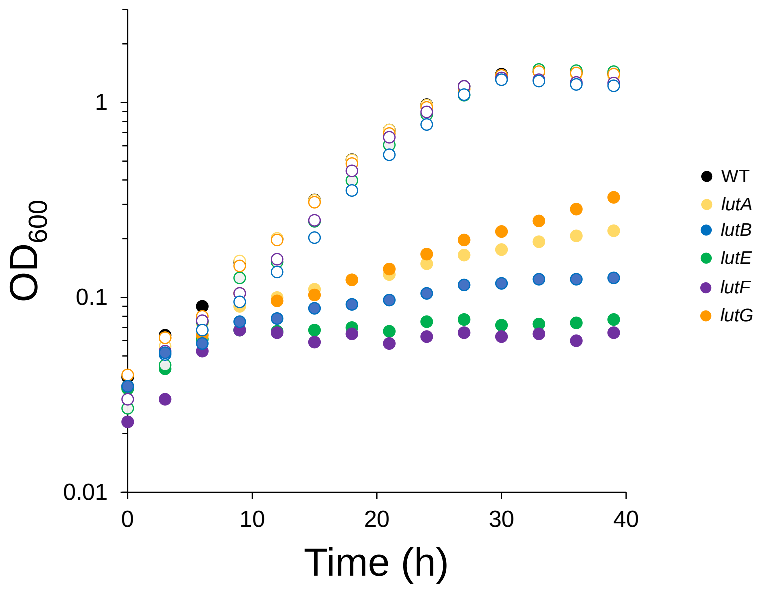

Fig S1. Complementation studies for the *lut* mutant strains. Graphs depict representative data from biological triplicates grown in methanol media (125 mM) containing 2 µM $LaCl_3$ and 5 µg/mL Tc. Filled circles represent wild type (WT) and mutant strains carrying the pCM62 empty vector. Empty circles represent each mutant strain with the respective gene cloned into pCM62.

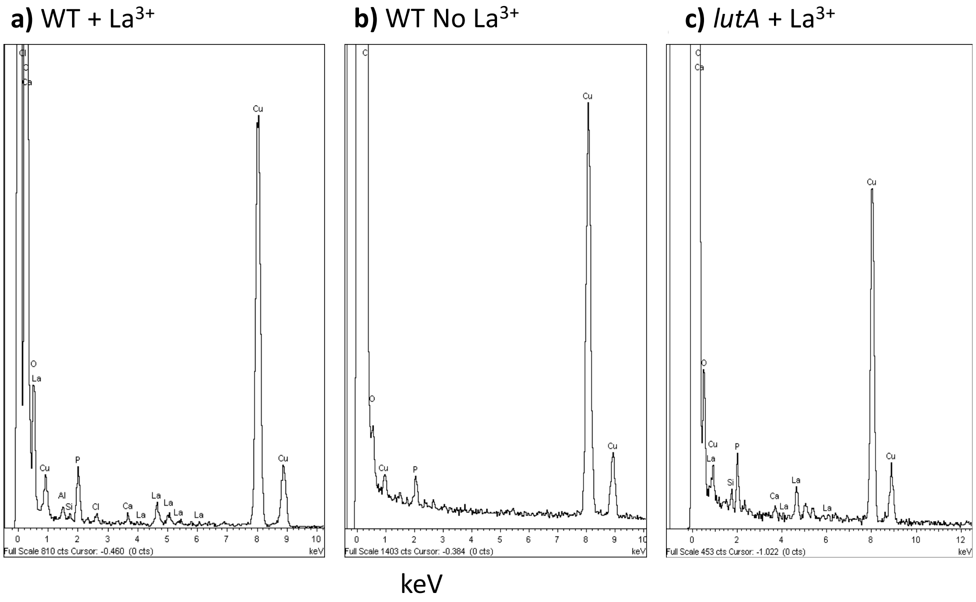

Fig S2. **Elemental analysis of cytoplasmic deposits.** Whole spectra of energy-dispersive X-ray analysis of the electron-dense deposits observed in the (a) cytoplasm of the wild-type (WT) strain grown with 20 µM $LaCl_3$ (b) cytoplasm of the wild-type strain grown without $LaCl_3$ and (c) periplasm of the *lutA* mutant strain grown with 20 µM $LaCl_3$. Microscopic images of these samples are depicted in Fig. 6 panels c and g and Fig. 7 panel f, respectively. Copper, carbon and silicon ion content is a result of these metals being present in the grids.
